# Interleukin-17A Triggers the Release of Platelet-Derived Factors Driving Vascular Endothelial Cells toward a Pro-Angiogenic State

**DOI:** 10.3390/cells10081855

**Published:** 2021-07-22

**Authors:** Aikaterini Gatsiou, Kateryna Sopova, Alexandros Tselepis, Konstantinos Stellos

**Affiliations:** 1 RNA Metabolism and Vascular Inflammation Group, Center of Molecular Medicine, Institute of Cardiovascular Regeneration, Johann Wolfgang Goethe University, 60596 Frankfurt am Main, Germany; aikaterini.gatsiou@ncl.ac.uk (A.G.); kateryna.sopova@newcastle.ac.uk (K.S.); 2 Laboratory of Clinical Biochemistry, Atherothrombosis Research Center, University of Ioannina, 45110 Ioannina, Greece; atselep@uoi.gr; 3 Biosciences Institute, Vascular Biology and Medicine Theme, Faculty of Medical Sciences, Newcastle University, Newcastle Upon Tyne NE1 3BZ, UK; 4 Department of Cardiology, Freeman Hospital, Newcastle Hospitals NHS Foundation Trust, Newcastle upon Tyne NE7 7DN, UK

**Keywords:** IL-17A, platelets, endothelial cells, angiogenesis, sprouting, VEGF

## Abstract

Platelets comprise a highly interactive immune cell subset of the circulatory system traditionally known for their unique haemostatic properties. Although platelets are considered as a vault of growth factors, cytokines and chemokines with pivotal role in vascular regeneration and angiogenesis, the exact mechanisms by which they influence vascular endothelial cells (ECs) function remain underappreciated. In the present study, we examined the role of human IL-17A/IL-17RA axis in platelet-mediated pro-angiogenic responses. We reveal that IL-17A receptor (IL-17RA) mRNA is present in platelets transcriptome and a profound increase is documented on the surface of activated platelets. By quantifying the protein levels of several factors, involved in angiogenesis, we identified that IL-17A/IL17RA axis selectively induces the release of vascular endothelial growth factor, interleukin -2 and -4, as well as monocyte chemoattractant protein -1 from treated platelets. However, IL-17A exerted no effect on the release of IL-10, an anti-inflammatory factor with potentially anti-angiogenic properties, from platelets. Treatment of human endothelial cell two-dimensional tubule networks or three-dimensional spheroid and mouse aortic ring structures with IL-17A-induced platelet releasate evoked pro-angiogenic responses of ECs. Our findings suggest that IL-17A may critically affect platelet release of pro-angiogenic factors driving ECs towards a pro-angiogenic state.

## 1. Introduction

Platelets are highly interactive components of the circulatory system with well-documented haemostatic properties. We have previously demonstrated the high clinical value of platelet activation for inflammatory diseases, including coronary artery disease, acute coronary syndrome, stroke, Alzheimer’s disease and atherosclerosis [1,2,3,4,5,6,7,8,9,10,11,12,13,14]. In vivo platelet depletion ameliorates atherothrombosis [15,16,17] whilst at the same time it impairs angiogenesis [18,19] and tissue regeneration [20,21,22,23]. Platelets modulate vascular and tissue inflammatory events either through cellular interactions [24,25,26,27,28,29,30,31,32] or through differential releasing of immunomodulatory ligands indirectly regulating the function of other blood and vascular wall cells [33,34]. We have previously shown that platelet-derived stromal cell-derived factor-1 (SDF-1) is critically involved in the chemotaxis and recruitment of blood cells to vascular endothelium controlling vascular inflammation and post-injury tissue healing [35,36,37,38]. Distinct populations of platelet alpha-granules can undergo selective release of different cytokines/chemokines. For instance, double immunofluorescence labelling of vascular endothelial growth factor (VEGF) (an angiogenesis stimulator) and endostatin (an angiogenesis inhibitor), or for thrombospondin-1 and basic fibroblast growth factor, confirms the segregation of stimulators and inhibitors into separate and distinct alpha-granules [19]. Treatment of human platelets with a selective protease-activated receptor -4, PAR4, agonist results in the release of endostatin-containing granules, but not VEGF-containing granules, whereas the selective PAR1 agonist liberates VEGF, but not endostatin-containing granules [19,39,40]. Apart from VEGF and endostatin, platelets contain and secrete a plethora of growth factors and chemokines that are involved in induction or inhibition of vascular regeneration and angiogenesis, like the well-characterized for its pro-angiogenic potency, the stromal derived factor 1 alpha (SDF1α). However, the exact mechanisms and key contributors of these important processes still remain unclear.

Increasing evidence shows that IL-17A is critically involved in atherosclerosis [41,42,43], autoimmune disease [44] and angiogenesis [44,45,46]. IL-17-producing T-helper (TH-17) cells are detected in prominent levels within atherosclerotic plaques of patients [43,47]. IL-17A is a potent proinflammatory cytokine that acts on fibroblasts, stromal, epithelial, endothelial cells, as well as on monocytes, stimulating the secretion of proinflammatory mediators, including chemokines like CXCL1, CXCL5, CXCL8 (IL-8), CXCL12 (SDF-1) and CCL2 (MCP1) [48,49]; cytokines, like tumour necrosis factor alpha (TNF-α), granulocyte colony-stimulating factor (GCSF) and interleukin 1-beta [48,49,50]. Interestingly, platelets bear the IL-17 receptor A (IL-17RA) on their surface [51] and sequester -among others- specific chemokines, including CXCL1, CXCL5, CXCL8, CXCL12 and CCL2 [52], which all have been reported to be regulated by IL-17A in various cell types.

Therefore, we hypothesized that expression of the IL-17RA on the surface of platelets may influence the secretion of platelet molecules with functional repercussions for the endothelial cell pro-angiogenic switch.

## 2. Materials and Methods

### 2.1. Preparation and Activation of Human and Murine Platelets 

Human blood was drawn from healthy donors after informed consent in compliance with the Declaration of Helsinki. Studies were approved by local authorities. For the flow cytometry studies, platelet-rich plasma (PRP) was prepared by centrifugation of 20 mL citrated blood drawn from healthy volunteers for 15 min at 128 g in room temperature (RT). PRP containing 1 × 10^6^ platelets was then incubated with ADP (50 μΜ, Sigma-Aldrich, St. Louis, Missouri, USA), PAR1-AP (PAR1-activating peptide, TRAP14, 50 μΜ, Sigma-Aldrich), PAR4-AP (100 μΜ, Sigma-Aldrich) or PBS for 10 min at RT. The surface expressions of IL-17 receptor A (IL-17RA-PE, monoclonal mouse IgG1 clone #133617; Cat#FAB177P, R&D Systems), GPIIb/IIIa (PAC-1-FITC, Cat#340507, BD Biosciences, San Jose, CA, USA) and P-selectin (CD62P-FITC, Cat#561922, BD Biosciences, San Jose, CA, USA) were analysed in the recorded 10,000 events using BD FACSCanto or FACSCalibur flow cytometer and the FACS Diva or Cellquest analysis software package (Becton-Dickinson, San Jose, CA, USA) with 1-color flow cytometry. For the permeabilization experiments, 2 × 10^8^ platelets were incubated with brefeldin A (Sigma-Aldrich, 20 μg/mL) and stimulated with PAR4-AP (100 μM) for 10 min in 37 °C followed by incubation with CD61-FITC (Cat#348093, BD Biosciences, San Jose, USA) for additional 15 min in room temperature at the dark. After a washing step (1% BSA in PBS), permeabilization was performed using the BD FACS™ Permeabilizing Solution followed by a washing step (1% BSA in PBS), the intracellular staining with the IL-17RA-PE antibody and an additional wash. For the isolation of purified platelets (washed platelets), platelets were isolated from platelet-rich plasma of six healthy young volunteers receiving no anti-platelet, anti-coagulant or anti-inflammatory medication, as previously described [53]. The same procedure was also followed for the isolation of the murine platelets [53]. Stimulations were performed in 1 mL of cell suspensions (2 × 10^9^/mL) with either human/murine recombinant IL-17A (50 ng/mL, R&D Systems), human PAR1-AP (100 μM) or PBS (resting platelets) for 10 min at 37 °C before collecting the releasate after a centrifugation of suspensions at 14,000× *g* for 5 min at 4 °C. Hundred microliter of each stimulated preparations (1/10 of the initially stimulated suspensions) were used for downstream applications unless otherwise specified.

### 2.2. RT-PCR Analysis of Highly Purified Platelet

For the isolation of platelet RNA, platelets were highly purified according to an established protocol [54]. Briefly, 40 mL of citrated blood was obtained by venipuncture from healthy volunteers and used for the generation of PRP. PRP was applied to a Sepharose column and gel filtered. Gel filtered platelets were washed 3 times in platelet wash buffer followed by a discontinuous iodixanol (Axis-Shield, Oslo, Norway) gradient centrifugation step. Highly purified platelets containing <0.00 leukocytes, validated by VetScan or flow cytometry analysis, were then used for the extraction of platelet mRNA with TRIzol^®^ (life technologies, Darmstadt, Germany), following the manufacturer’s recommendation. Peripheral blood mononuclear cells were isolated from 40 mL of citrated blood of healthy volunteers by density gradient centrifugation with Ficoll-Paque^®^ (GE-Healthcare Bio-Sciences AB, Uppsala, Sweden) and lysed for mRNA extraction with TRIzol^®^ according to the manufacturer’s instructions. The extracted RNA was reverse transcribed into cDNA and the cDNA was amplified with primers specific for IL-17RA (87-bp product); sense sequence: GTGCTGTCGCCACCAAGTGC and anti-sense sequence: GGGCAGGAAACAGTCGCGGAG. Additionally, amplification of CD41 and CD45 mRNA was used as quality control of samples. The presence of the mRNA of IL-17RA in platelet loading was verified by the existence of an 87-bp length product in the electrophoresis gel.

### 2.3. Western Blot

For Western blot analysis, platelets were lysed with 100 μL lysis buffer (20 mM Tris, pH 7.4, 150 mM NaCl, 1 mM ethylenediaminetetraacetic acid, 1 mM ethyleneglycoltetraacetic acid, 1% Triton, 2.5 mM sodium pyrophosphate, 1 mM β-glycerophosphate, 1 mmol Na_3_VO_4_, 1 μg/mL leupeptin, 1 mM phenylmethylsulfonyl fluoride, and 10 mmol NaF) for 15 min on ice. After centrifugation for 15 min at 14,000× *g* (4 °C), the protein content of the samples was determined according to the Bradford method. Proteins were loaded onto sodium dodecyl sulphate–polyacrylamide gels and blotted onto polyvinylidene difluoride membranes (Millipore, Schwalbach, Germany). Western blots were performed by using antibodies directed against human IL-17RA (anti-human-IL-17RA, R&D Systems, monoclonal mouse IgG_1_ clone # 133617; Cat#MAB177; 1 μg/mL) and α-tubulin (1/1000; Dianova, Hamburg, Germany). Enhanced chemiluminescence was performed according to the manufacturer’s instructions (Amersham Biosciences, Freiburg, Germany).

### 2.4. Light Transmission Aggregometry

Per cent platelet aggregation after stimulation with 10 µM PAR1-AP, 10 µM PAR4-AP, 0.02 U/mL Thrombin (all from Sigma Aldrich) or 1 µg/mL Collagen (BD Biosciences, San Jose, CA, USA) in combination or not for 2 min or 1 h with 50 ng/mL human recombinant IL-17A was assessed with the turbidimetric method using a Chronolog Lumi aggregometer with Aggro-Link Software.

### 2.5. Protein Quantification in Platelet Releasates

The levels of all pro- or anti- inflammatory factors, but SDF1α, were quantified in platelet releasates using a custom multiplexed antibody-based assay (Q-Plex ELISA, Quansys Biosciences, 365 North 600 West Logan, UT 84321, USA). The SDF1α levels were recorded using a Quantikine ELISA (R&D Systems, Minneapolis, MN, USA). All measurements were performed according to the manufacturer’s instructions. The absolute quantification (pg/mL) was enabled through reference of absorbance/chemiluminescence readings with the generated standard curve per assay.

### 2.6. Capillary Tube Formation Assay

HUVECs (12 × 10^4^), inoculated with either VEGF, IL-17A (supplied in platelet suspension buffer to correspond to the residual levels of the cytokine in the platelet supernatant following the stimulation of platelets; 5 ng/mL) or stimulated platelet releasate from 2 × 10^8^/mL, as indicated, were cultured in a 12-well plate (Greiner, Kremsmünster, Austria) coated with 200 μL Matrigel Basement Membrane Matrix (BD Biosciences, San Jose, CA, USA), as we have previously described [55]. Tube length was quantified after 24 h by measuring the cumulative tube length in 10 random microscopic fields with a computer-assisted microscope using Axiovision 4.5 (Zeiss, Jena, Germany). Tube length was quantified after 24 h in culture by measuring the cumulative tube length in five random microscopic fields with a computer-assisted Axiovert 100 M inverted microscope equipped with a Plan-NEOFLUAR objective (5×/0.30; Carl Zeiss, Jena, Germany) and analysing the images using Axiovision 4.9.1 software (Carl Zeiss, Germany). The mean tube length of the five random microscopic fields per condition and per experiment was calculated.

### 2.7. 3-D Sprouting Assay

Endothelial cell spheroids were generated as previously described [55] and incubated with either IL-17A (supplied in platelet suspension buffer to correspond to the residual levels of the cytokine in the platelet supernatant following the stimulation of platelets; 5 ng/mL) or treated platelet supernatant as indicated. In vitro angiogenesis was quantified using an Axiocam MR digital camera with an Axiovert 100 M inverted microscope equipped with a Plan-NEOFLUAR objective (10×/0.30; Carl Zeiss, Jena, Germany). Quantification was performed by measuring the cumulative length of the sprouts that had grown out of each spheroid using a digital imaging software (Axioplan, Zeiss, Jena, Germany) and analysing 10 spheroids per group and experiment. The mean of the 10 spheroids per condition and per experiment was calculated.

### 2.8. Ex Vivo Murine Aortic Ring Assay

In order to evaluate the in vivo potential of our findings, we employed the physiological relevant ex vivo angiogenesis assay, which has been previously described in detail [56]. Briefly, littermate 8–12-week-old C57BL/6 mice were humanely sacrificed according to the principles of laboratory animal care and German laws. The aorta was carefully removed from each mouse and cleaned under a stereoscope from the extraneous fat, tissue and branching vessels to avoid blunted angiogenic responses. Next, the aorta was flushed to remove the residual blood from the lumen by a 1 mL syringe filled with HBSS (Sigma-Aldrich) and cut into rings of ~1 mm width, which were preserved in HBSS for short time till processing. A collagen type I-based gel (1 mg/mL; pH = 7.4) was prepared on ice (to avoid premature polymerization) and 60 μL of the gel-mixture were dispensed into each well of a 96-flat bottom dish (Eppendorf) on ice. With the help of forceps, the rings were gently embedded inside the non-polymerized gel ensuring that one ring corresponds to one well and that the luminal axis of the ring is placed parallel to the bottom of the well. After 1 h incubation at 37 °C (5% CO_2_), every ring received 150 μL culture medium consisting of EBM (Lonza; without growth factors) and Pen/Strep and murine platelet releasate stimulated with murine recombinant IL-17A (50 ng/mL) or PBS. Aortic rings from the same mouse aorta were used for the comparison of the different stimuli to overrule any genetic background effect. The aortic rings were incubated for seven days with two medium changes in between. The aortic ring sprouts were visualized by staining of the aortic rings with iB4 (marker for endothelial cells) and mosaic images were acquired by using an Axiocam MR digital camera with an Axiovert 100 M inverted and computer assisted-epifluorescence microscope (5× objective; AxioVert Zeiss, Carl Zeiss, Jena, Germany). The mean length of three rings per condition and per experiment was calculated using the ImageJ software.

### 2.9. Statistical Analysis

Normal distribution of variables was tested by Shapiro–Wilk and Kolmogorov–Smirnov tests. Continuous variables are presented as mean (SEM) and compared with the use of Student’s *t* test when normally distributed, and as median (IQR) compared with two-tailed Mann–Whitney U test when non-normally distributed. For comparison of more than 2 groups one-way ANOVA or the non-parametric Kruskal–Wallis were used. Details for the specific statistical analysis of each experiment are provided in the corresponding figure legends. Statistical significance was deemed at *p* < 0.05. Statistical analysis was performed using GraphPad Prism 7.

## 3. Results

### 3.1. IL-17RA Is Expressed on Platelet Surface and Induced upon Platelet Activation

**Figure 1:**
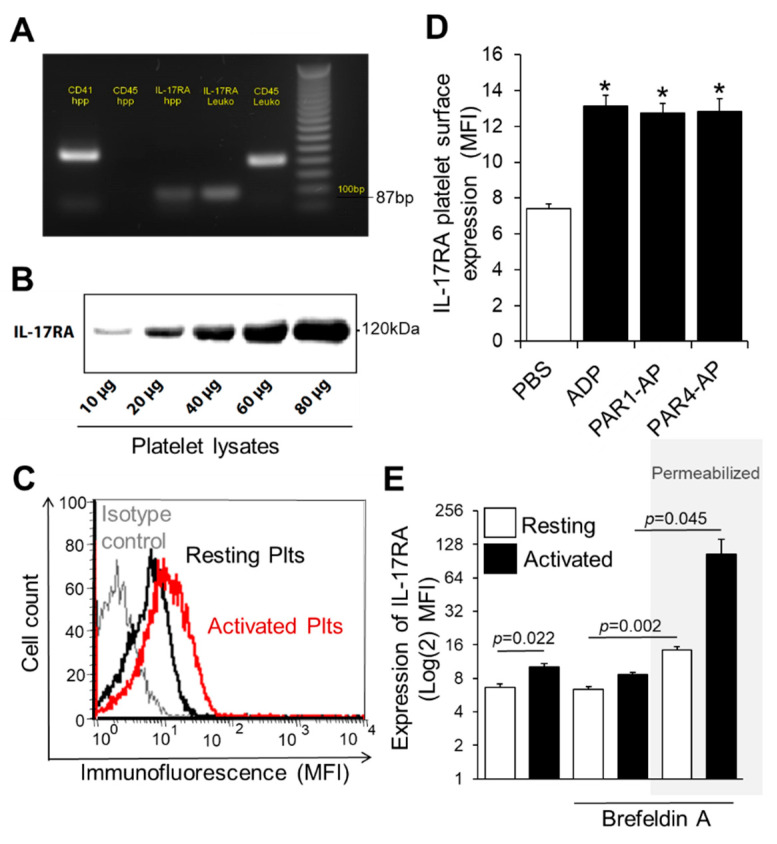
Expression of IL-17RA on platelet surface is increased in activated platelets. (**A**) Total RNA was isolated from highly purified platelets and reverse transcribed into cDNA. The cDNA was amplified with primers specific for IL-17RA (87-bp product). Additionally, amplification of CD41 and CD45 mRNA was used as quality control of samples. A 100-bp DNA ladder is shown on the left lane. (**B**) Western blotting analyses of IL-17RA. The presence of IL-17RA was confirmed in lysates from resting platelets. (**C**) A representative overlay histogram for IL-17RA expression in washed platelets. Human washed platelets were activated with ADP (red line) or PBS (resting platelets, black line) and stained with a monoclonal antihuman anti-IL-17RA antibody (PE-anti-IL-17RA; red line) or with control isotype antibody (PE-conjugated IgG_1_) (grey line). (**D**) Platelet-rich plasma (PRP) was stimulated with ADP (50 μΜ), TRAP14 (50 μΜ), PAR4-AP (100 μΜ) or PBS for 10 min at room temperature. Platelet suspension was then stained with PE-anti-IL-17RA or with PE-conjugated IgG_1_ and analysed with flow cytometry. Values are shown as Mean MFI ± SEM of three independent experiments (* *p* < 0.05 vs. PBS). With regard to statistics, *p*-value is <0.05 for all comparisons indicated as determined by one-way ANOVA followed by Dunnett’s multiple comparison test. (**E**) Washed platelets were activated with PAR4-AP (100 μM) or PBS in the presence or absence of Brefeldin A (BFA; 20 μg/mL), an inhibitor of protein transport, and labelled with PE-anti-IL-17RA or PE-conjugated IgG_1_. In some experiments, platelets were first permeabilized before staining. Values are shown as Mean MFI ± SEM of three independent experiments. *p*-value reported derived from pairwise comparisons using two tailed *t* test. One-way ANOVA followed by Bonferroni’s multiple comparison test reports *p* = 0.003 for the permeabilized platelets prior treated with BFA and PAR4-AP compared to non-permeabilized platelets that received similar treatment.

In order to investigate the IL-17RA expression on platelets, we first studied the presence of IL-17RA mRNA in platelet transcriptome using RT-PCR to investigate whether IL-17RA is part of the platelet transcriptome. For this purpose, highly purified platelets were used specifically in order to avoid potential contamination resulting in false positive results. The presence of the mRNA of IL-17RA in platelet loading was confirmed by the existence of an 87-bp length product in the electrophoresis gel (Figure 1A). Additionally, with the help of Western blot analysis, we were able to confirm the presence of IL-17RA protein in platelet lysates (Figure 1B). Platelet surface expression of IL-17RA was determined by flow cytometry in platelets stimulated with ADP, TRAP14 or PAR4-AP as well as in platelets treated with PBS (resting). Interestingly, we observed that platelet activation significantly increases the surface expression of IL-17RA when compared with PBS (*p* < 0.05 vs. PBS, Figure 1C,D). Moreover, we found that intraplatelet levels of IL-17RA are significantly increased after platelet stimulation with PAR4-AP or PBS when comparing with intact platelets activated in the presence of brefeldin A, an inhibitor of protein transportation from endoplasmic reticulum to Golgi apparatus, as measured by flow cytometry (*p* = 0.002; resting intact vs. permeabilized platelets; *p* = 0.045; PAR4-AP intact vs. permeabilized platelets; Figure 1E), suggesting that the source of the increased surface IL-17RA occupancy was the platelets. These findings together indicate that IL-17A may play an important role for platelet function.

### 3.2. IL-17A Has No Effect over Platelet Aggregation

Next, we investigated whether the IL-17A/IL17-RA axis may exert any effect on platelet aggregation, an important function of these cells. To gain first insights, platelets were incubated with IL-17A and the surface expression of the open conformation of glycoprotein IIb/IIIa (GPIIb/IIIa; PAC-1) as well as the P-selectin (CD62P) levels were quantified by flow cytometry. Interestingly, IL-17A stimulated-platelets exhibited unchanged levels in either GPIIb/IIIa activation or P-selectin expression (Supplementary Figure S1A,B). In order to directly address the role of IL-17A in platelet aggregation, the levels of platelet aggregates were recorded using light transmission following incubation of IL-17A alone for 2 min or in combination with platelet agonists (thrombin, PAR1-AP, collagen and PAR4-AP) for 2 min or 1 h to allow sufficient time for potential interactions. Concordantly with the lack of effect on activation of GPIIb/IIIa, IL-17A addition to platelets did not influence the aggregatory capacity of platelets in any of the tested settings (Supplementary Figure S2C). We then investigated whether increased or decreased doses of IL-17A alone (10–200 ng/mL) may affect platelet aggregation. Despite the induction of platelet aggregation in response to the stimulation of platelets with established agonists, no effect was documented with regard to IL-17A-stimulated platelets (Supplementary Figure S3A). We also tested whether co-incubation of platelets with IL-17A and different doses of ADP (2–10 μM) would influence the aggregatory potency of platelets. No differences were observed in this experimental setting either (Supplementary Figure S3B).

These findings suggest that IL-17A/IL-17RA axis is redundant for platelet aggregation.

### 3.3. IL-17A Triggers Release of Several Platelet-Derived Factors with Pro-angiogenic Potential

**Figure 2:**
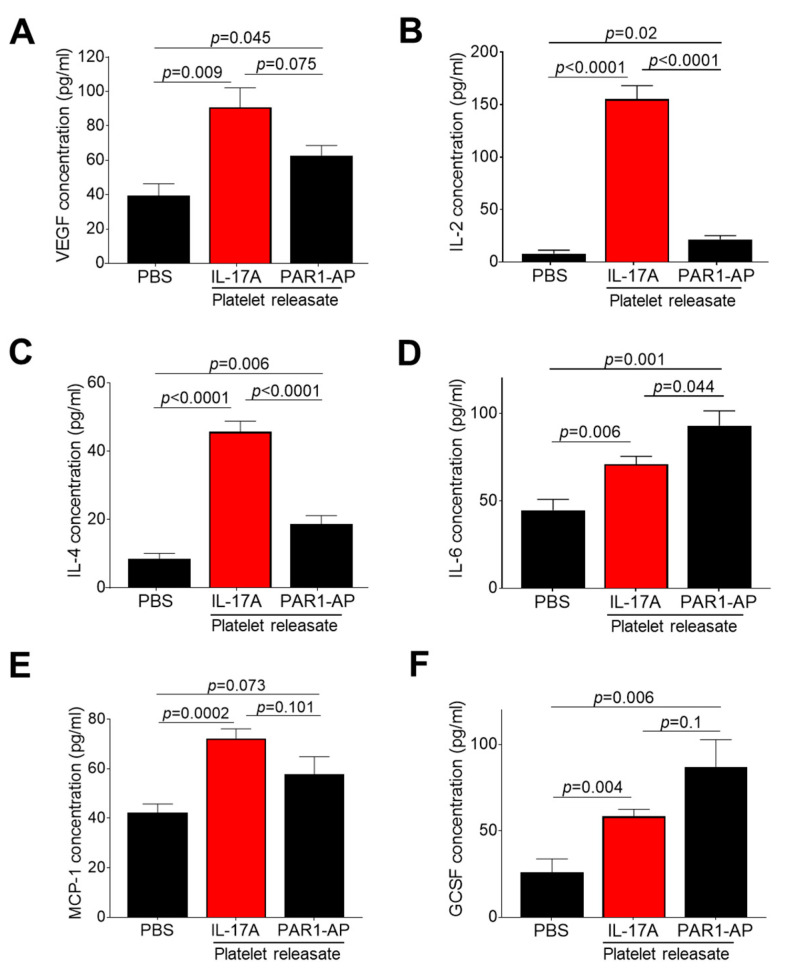
IL-17A/IL-17RA axis mediates the release of several platelet-derived factors with dual function in both inflammation and angiogenesis. Washed platelets were incubated with IL-17A (50 ng/mL) or PAR1-AP (100 μΜ) or PBS (resting platelets) for 10 min at 37 °C. Using ELISA, the recovered releasates were then analysed for (**A**) vascular endothelial growth factor (VEGF), (**B**) interleukin-2 (IL-2), (**C**) interleukin-4 (IL-4), (**D**) interleukin-6 (IL-6), (**E**) monocyte chemoattractant protein-1 (MCP-1) and (**F**) granulocyte colony-stimulating factor (GCSF). Values are presented as the mean concentration (pg/mL) ± SEM of six independent experiments. With regard to statistics, *p*-value of two-tailed *t* test is reported per indicated pairwise comparison as normality test was passed. Adjusted *p*-value for IL-17A-treated releasate protein levels (vs. PBS-treatment) is *p* < 0.05 for all as determined by one-way ANOVA followed by Dunnett’s multiple comparison test. No statistical significance is denoted as n.s.

Based on the fact that IL-17A promotes angiogenesis by inducing the secretion of known molecules with pro-angiogenic role, in pericytes, macrophages and endothelial cells [57], we hypothesized that platelets treated with IL-17A may secrete increased levels of inflammatory mediators with angiogenic potential. For this purpose, we determined the protein levels of 11 factors, all known to be secreted by platelets and have a part in angiogenesis, in resting and in IL-17A-stimulated platelet releasate. While stromal-derived factor 1α (SDF1α), angiopoietin-2 (Ang-2), interleukin-8 (IL-8), platelet-derived factor (PDGF), C-X-C motif chemokine ligand 1 (CXCL1) and chemokine-ligand 5 (CCL5) were not significantly enriched in IL-17A-stimulated platelet releasates (Supplementary Figure S3A–G), the levels of VEGF, IL-2, IL-4, IL-6, monocyte chemoattractant protein-1 (MCP-1), and GCSF were profoundly increased in releasates derived from platelets incubated with IL-17A compared to the one from resting platelets (PBS) (*p* < 0.05 for all vs. PBS-induced platelet releasate, Figure 2A–F). Of note, the increase of VEGF, IL-2, IL-4, and MCP-1 levels in response to IL-17A compared to the corresponding levels observed in PAR1-AP-treated releasates exhibited either a noticeable trend (for VEGF: IL-17A vs. PAR1-AP releasates: *p* = 0.075; Figure 2A) or a significant difference (for IL-2, IL-4, MCP-1: IL-17A vs. PAR1-AP releasates: *p* < 0.05 for all; Figure 2B,C,E), while PAR1-AP stimulation effected more drastically the release of SDF1α, IL-8, CXCL1 and CCL5 from platelets (PAR1-AP vs. PBS: *p* < 0.05 for all; Supplementary Figure S3A,C,F,G). Contrastingly, neither IL-17A nor PAR1-AP stimulation of platelets elicited the secretion of an anti-inflammatory factor known to exert anti-angiogenic properties, we spiked in the multiplexed antibody-based assay, IL-10 (vs. PBS-induced platelet releasate: *p* > 0.05 for all; Supplementary Figure S3D,H). These findings highlight the importance of the human IL-17A/IL-17RA axis in platelet release of *bona fide* pro-angiogenic factors, like VEGF, or inflammatory mediators with dual function, both in inflammation and angiogenesis, like IL-2 and IL-4.

### 3.4. Releasate of IL-17A-Induced Platelets Has an Overall Pro-angiogenic Potential

**Figure 3:**
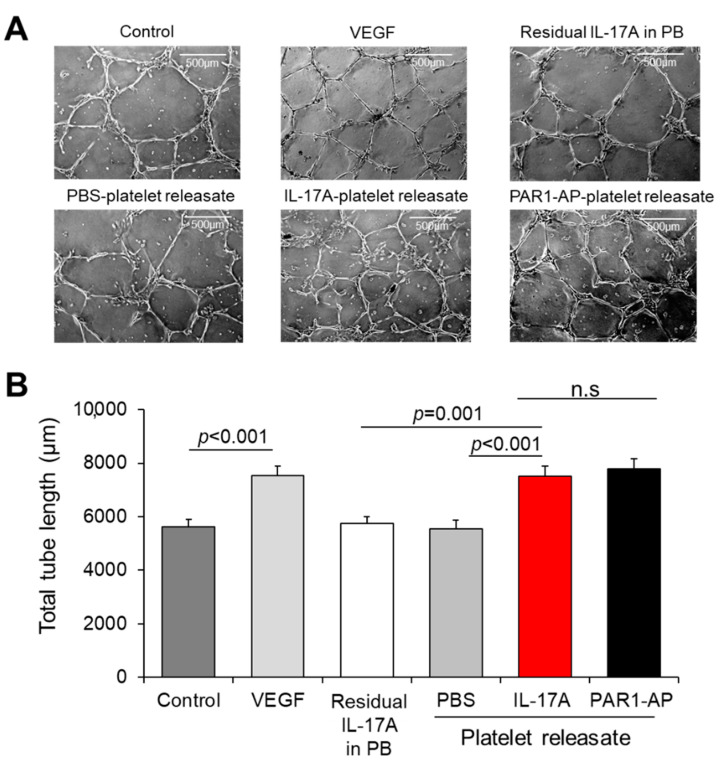
IL-17A-treated platelet releasate promotes endothelial cell tube formation. (**A**) Capillary tube formation in Matrigel-coated wells following exposure of human umbilical vein endothelial cells (HUVECs) to VEGF (10 ng/mL), residual IL-17A alone in platelet buffer (at a final concentration of 5 ng/mL) or to releasate from platelets prior incubated with IL-17A (50 ng/mL), PAR1-AP (100 μΜ) or PBS (resting platelets) for 18 h. (**B**) Quantification of cumulative tube length was determined in 10 non-overlapping fields. Values are presented as the mean of total tube length (μm) ± SEM of three independent experiments. With regard to statistics, adjusted *p*-value as determined by one-way ANOVA and Bonferroni’s multiple comparison test is reported per indicated comparison. No statistical significance is denoted as n.s. Scale bars indicate 500 μm.

After demonstrating that IL-17A increases the release of inflammatory molecules from platelets involved in angiogenesis, we were prompted to assess the potential of IL-17A-induced platelet releasate to drive endothelial cells towards a pro-angiogenic state. We tested our hypothesis using two in vitro EC angiogenesis models: (a) capillary-like tube formation network and (b) sprouting spheroid assay. Platelets from healthy volunteers were isolated and either allowed to rest (PBS treatment) or treated with IL-17A or PAR1-AP. The recovered releasate was directly administered to 2D endothelial network structures (Figure 3A). IL-17A-treated platelets releasates evoked significantly increased total tube length of EC network in comparison to resting platelets or to the corresponding residual quantity of IL-17A alone dissolved in platelet buffer (PB) without platelets (IL-17A-treated releasate: 7511 ± 371 μm vs. PBS-treated releasate: 5537 ± 346 μm or vs. IL-17A in PB: 5746 ± 258 μm; *p* < 0.001 and *p* = 0.001, respectively; Figure 3B). Notably, IL-17A-treated platelet releasate exert augmented capillary tube formation levels similar to those induced directly by the main proangiogenic regulator, VEGF (VEGF vs. untreated ECs: 7540 ± 354 μm vs. 5623 ± 273 μm, *p* < 0.001, Figure 3B). The total tube length of the network was also comparably extended when ECs received the releasate derived from platelets activated with PAR1-AP, an established platelet agonist, compared to PBS-treated platelet releasate treatments (*p* < 0.05, Figure 3B). In line with the documented pro-angiogenic potential of IL-17A, direct administration of IL-17A to endothelial cells increased the tubule formation compared to PBS-treated cells (Supplementary Figure S4A,B).

**Figure 4:**
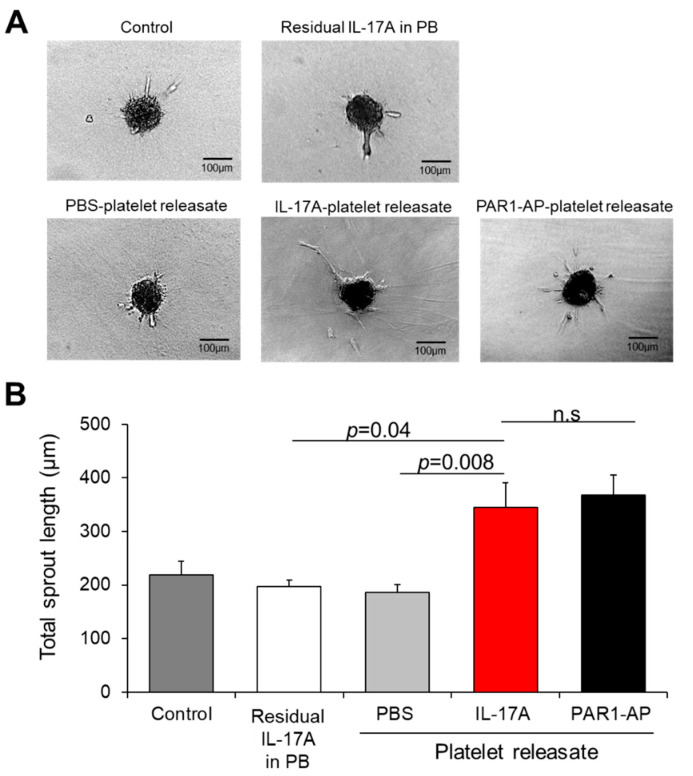
IL-17A platelet releasate triggers sprouting of endothelial cells. (**A**) Endothelial cell spheroids embedded into collagen gel were incubated with medium (untreated ECs), residual IL-17A alone in platelet buffer (at a final concentration of 5 ng/mL) or releasate from platelets induced with IL-17A (50 ng/mL), PAR1-AP (100 μΜ) or PBS (resting platelets) for 24 h. One representative picture of each group is shown. (**B**) Quantitative analysis of the mean of the total sprout length of at least ten spheroids per experimental group and per experiment was performed. Data are shown as mean of the total sprout length (μm) ± SEM of six independent experiments. With regard to statistics, adjusted *p*-value as determined by one-way ANOVA and Bonferroni’s multiple comparison test is reported per indicated comparison. No statistical significance is denoted as n.s. Scale bars indicate 100 μm.

In order to interrogate the pro-angiogenic potential of IL-17A platelet releasate during the sprout formation, which is a critical step of angiogenesis [58], we employed the 3D in vitro angiogenesis model that comprises collagen gel-embedded spheroids of ECs. For this purpose, platelet releasates triggered by IL-17A, PAR1-AP or PBS (as a control) were administered to spheroids (Figure 4A). We recorded an increase in the overall sprout length of the spheroids treated with IL-17A-platelet releasate (345 ± 46 μm) when compared to PBS-treated supernates (vs. 185 ± 14 μm, *p* = 0.008; Figure 4B) or to the respective quantity of the residual IL-17A in platelet buffer without platelets (vs. 197 ± 11 μm, *p* = 0.037; Figure 4B). Of interest, PAR1-AP-induced releasates promoted the sprout formation to a similar extent as IL-17A-treated supernates (368 ± 36 μm), given that no statistically significant difference was documented among the two conditions (Figure 4B).

**Figure 5:**
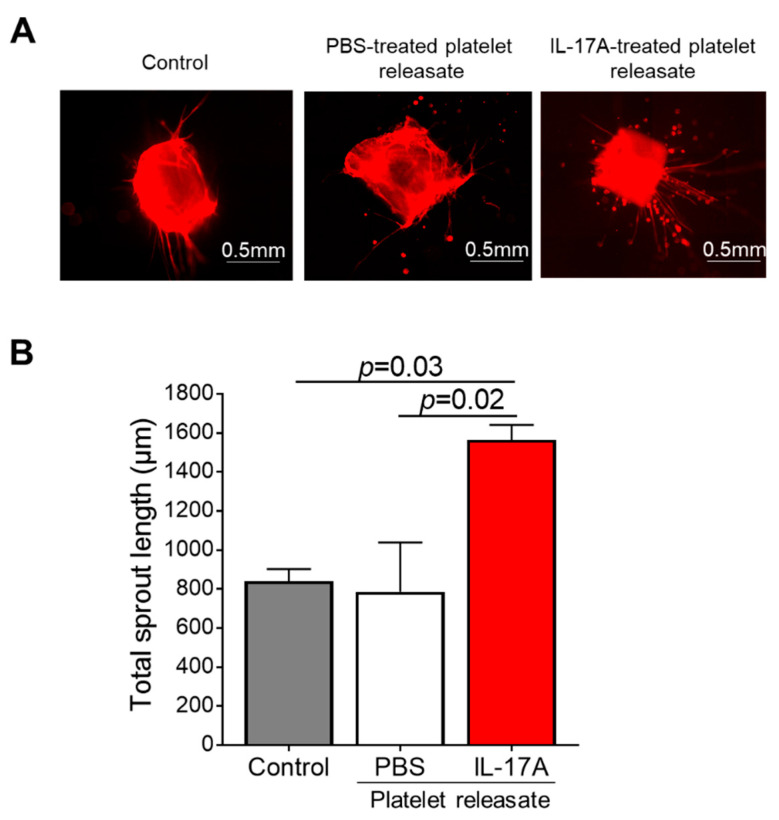
Murine IL-17A platelet releasate triggers aortic ring microvessel sprouting. (**A**) Aortic rings of approximately 1mm long embedded into collagen gel were incubated with medium (untreated rings; Control) or releasate from platelets prior stimulated with IL-17A (50 ng/mL) for 7 days. One representative picture of each group is shown. (**B**) Quantitative analysis of the mean total sprout length of three rings per experimental group and per experiment was performed. Data are shown as mean of the total sprout length (μm) ± SEM of three independent experiments. With regard to statistics, adjusted *p*-value as determined by one-way ANOVA and Dunnett’s multiple comparison test is reported per indicated comparison. Scale bars indicate 0.5 mm.

In order to seek further confirmation with regard to the validity of our findings, we utilized the murine 3D aortic ring assay which closely resembles the in vivo angiogenic responses in a similar timescale. IL-17A-treated murine platelet releasate boosted the isolectin B4-stained microvessel sprouting compared to either the PBS-treated releasate (1564 ± 78 vs. 786 ± 252 μm, *p* = 0.02) or untreated aortic rings (1564 ± 78 vs. 840 ± 62 μm, *p* = 0.03; Figure 5A,B). 

Collectively, these results support the pro-angiogenic potential that the human and murine IL-17A/IL-17RA axis confers to platelet releasate.

## 4. Discussion

Our study demonstrates: (a) the presence of IL-17RA mRNA in platelets, and the increase of IL-17RA surface expression on the platelet membrane in response to platelet activation; (b) IL-17A/IL17RA axis has no effect on platelet aggregation; (c) IL-17A induces the release of platelet-derived molecules, including VEGF; and (d) upon treatment of endothelial cells (ECs) with IL-17A-induced platelet releasate, the angiogenic capacity of ECs is profoundly increased as shown in both human and murine angiogenic assays in vitro and ex vivo. 

IL-17RA is expressed in several cell types and its protein is known to be part of platelet lysate [51]. Our study shows that IL17RA mRNA is present in platelet transcriptome and that the intracellular expression levels of IL-17RA are increasing in activated platelets leading to the higher occupancy of the platelet membrane. Protein synthesis in platelets has been reported to be feasible within 10–30 min [59,60], likely attributed to fact that platelet translation starts with a readily available template (mRNA). Our settings involved stimulation of platelets for a total period of time of up to 25 min. For our experiments to quantify the intracellular levels of IL-17RA, we have used brefeldin A that is known to inhibit the protein trafficking from endoplasmic reticulum to Golgi. Brefeldin A has been also shown to act as the most effective platelet microparticle inhibitor [61]. A limitation of our flow cytometry experiments is that our approach cannot fully exclude the presence of potentially aggregated platelets after stimulation of platelets with agonists. Nevertheless, we believe that this is unlikely since whole blood was drawn in the presence of citrate anticoagulation. These findings together pinpoint towards that either newly surfaced IL-17RA protein molecules may be synthesized or released from microparticle compartments upon platelet activation. However, future studies employing biochemical assays shall elucidate the exact underlying mechanisms by which this increase occurs as well as the exact kinetics. 

An important function of platelets is their signature feature to aggregate and facilitate haemostasis. We report that IL-17A/IL-17RA axis is dispensable for platelet aggregation. This finding is further consolidated by the unchanged levels of the surface abundance of the opened conformation of GPIIb/IIIa. A previous study has reported that co-treatment of platelets with a high dose of IL-17A and low dose of ADP triggers a 40% increase in the aggregatory capacity of platelets [51]. We have also tested a similar setting by incubating platelets with a high dose of IL-17A and different doses of ADP, including low dose of ADP. We did not register any differences in the aggregation. The same study though did not report any direct effects of IL-17A alone in platelet aggregation.

Platelets, in addition to maintaining haemostasis, play a critical role in regulating angiogenesis by releasing factors that promote the growth of new vessels [19,39,62]. Several studies suggest that IL-17A has also an important role in angiogenesis by enhancing the secretion of pro-angiogenic molecules from fibroblasts and other cells, which in turn induce the angiogenic capacity of endothelial cells [45,46,63]. Platelets interaction with endothelial cells is a highly dynamic process the outcomes of which depend on microenvironmental cues [64]. Due to the prominent role of IL-17A in angiogenesis, we investigated whether it may direct the release of platelet content influencing EC to adopt a pro-angiogenic status, a key switch of vascular endothelial cells towards angiogenesis. Prior reports suggest that pro-angiogenic factors are predominantly enriched within the platelet content [19,40]. As such, we selected to quantify eleven factors, among them, SDF1α and VEGF which critically affect endothelial cell angiogenesis [65,66]. Our results reveal that IL-17A triggers the release of VEGF, but not of SDF1α. Nevertheless, PAR1-AP-activation of platelets released massive amounts of SDF1α in our experiments which are in agreement with previous investigations [39]. We shall note here that to our surprise, we observed an almost selective induction of VEGF, IL-2, IL-4 and MCP-1 release from platelets treated with IL-17A at higher levels than a potent platelet agonist, PAR1-AP, despite the latter being used at much higher concentration (100 μM) compared to IL-17A (50 ng/mL) in our experimental settings. Whether the release of these molecules is governed by different intracellular signalling pathways which could explain these differences remains to be examined by future studies. Future efforts are also warranted to elucidate the pathways that are involved in the secretion of the IL-17A-dependent factors. Of particular interest, the levels of IL-10, an anti-inflammatory cytokine with anti-angiogenic potential [67], did not exhibit any fluctuations within the platelet releasate of IL-17A treated platelets compared to control treatment. In view also of the findings observed in the angiogenic assays employed in our study, we suggest that the resultant of the IL-17A-induced platelet proteome has a pro-angiogenic potency. Nevertheless, large scale proteomics investigations are required to define the landscape of the IL-17A-induced platelet secretome and the balance between pro- and anti- angiogenic factors release. Neutralization experiments targeting for example VEGF could provide further insights into the dissection of the exact contribution of each IL-17A-dependent platelet released factor to angiogenesis.

The “sponging” nature of platelets, enabling them to absorb various molecules from the microenvironment, precludes any definitive conclusions with regard to the *bona fide* platelet origin of the released molecules. In this study, we chose to study factors the presence of which has been recorded in either the platelet proteome, transcriptome or both, such as VEGF [68,69]. We may accordingly speculate that IL-17A could increase the synthesis of VEGF based on the mRNA molecules of the platelet transcriptome. The exact molecular mechanism though remains to be explored by future investigations. 

In conclusion, our findings suggest that the pro-inflammatory cytokine, IL-17A, modulates the platelet-mediated endothelial cell angiogenesis. Among the IL-17A-induced secreted factors we identified in the present study, several of them, like MCP-1, IL-2, and IL-4, exert a dual function in angiogenesis but also in inflammation. Therefore, our study adds a new pro-angiogenic dimension to the pro-inflammatory function of IL-17A highlighting the central role of platelets in the cross-road of inflammation and angiogenesis. Future efforts are anticipated to therapeutically explore the potential of our findings in diseases marked by increased IL-17A levels and platelet reactivity or angiogenic responses, such as rheumatoid arthritis, atherosclerosis, psoriasis, cancer and myocardial infarction.

## Data Availability

The data presented in this study are available upon reasonable request from the corresponding author.

## Supplementary Materials

The following supporting information is available online at https://www.mdpi.com/article/10.3390/cells10081855/s1, Supplementary Figure S1: No effect of IL-17A alone or in combination with thrombin, PAR1-AP, collagen or PAR4-AP in platelet aggregation. Supplementary Figure S2. No effect of IL-17A alone in different doses or in combination with different doses of ADP in platelet aggregation. Supplementary Figure S3. Pro- and anti-inflammatory factors that do not exhibit different levels in the platelet releasate following IL-17A stimulation of platelets. Supplementary Figure S4. Direct effect of IL-17A on capillary-like tube formation of endothelial cells.
