# Supplementary material for: Interleukin-17A Triggers the Release of Platelet-Derived Factors Driving Vascular Endothelial Cells toward a Pro-Angiogenic State
Source: Cells. 2021 Jul 22;10(8):1855. doi: 10.3390/cells10081855 (PMC8392697; doi:10.3390/cells10081855)
Supplement: Supplementary file 1 [file cells-10-01855-s001.zip › cells-1289913-supplementary.pdf]

# Supplementary Materials

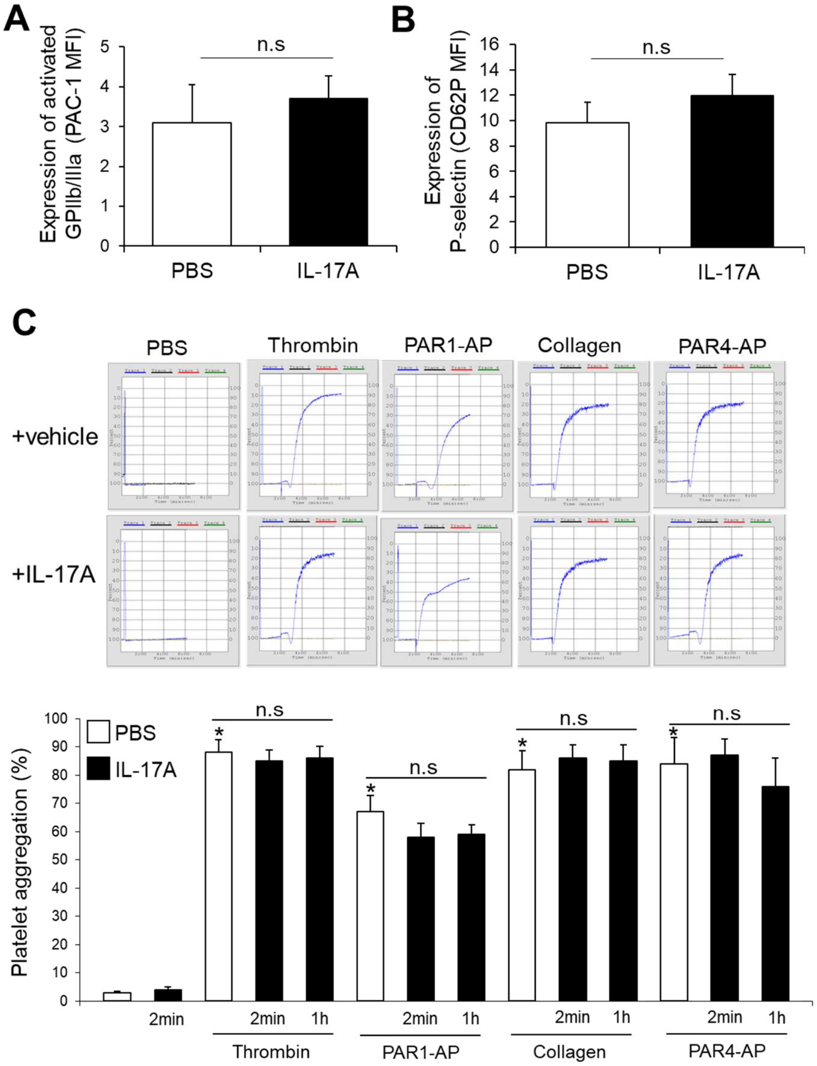


**Figure S1.** No effect of IL-17A alone or in combination with thrombin, PAR1-AP, collagen or PAR4-AP in platelet aggregation. (**A**–**B**) Expression of the open conformation of GPIIb/IIIa (PAC-1) (A) or P-selectin (CD62P) (B) on the surface of IL-17A (50 ng/ml) stimulated platelets. **C**) Aggregation of IL-17A-stimulated platelets for 2 min at 37oC or co-stimulated with thrombin, PAR1-AP, collagen or PAR4-AP for 2 min at 37oC or 1 h at room temperature. Representative images from the platelet aggregation experiments using light transmission aggregometry (upper panel) and quantification as percentage of aggregation (%) from three independent experiments (lower panel). Data are presented as mean±SEM. With regards to statistics, *P-value is determined by one-way ANOVA followed by Bonferroni's multiple comparison test is reported per indicated comparison. No statistical significance is denoted as n.s.

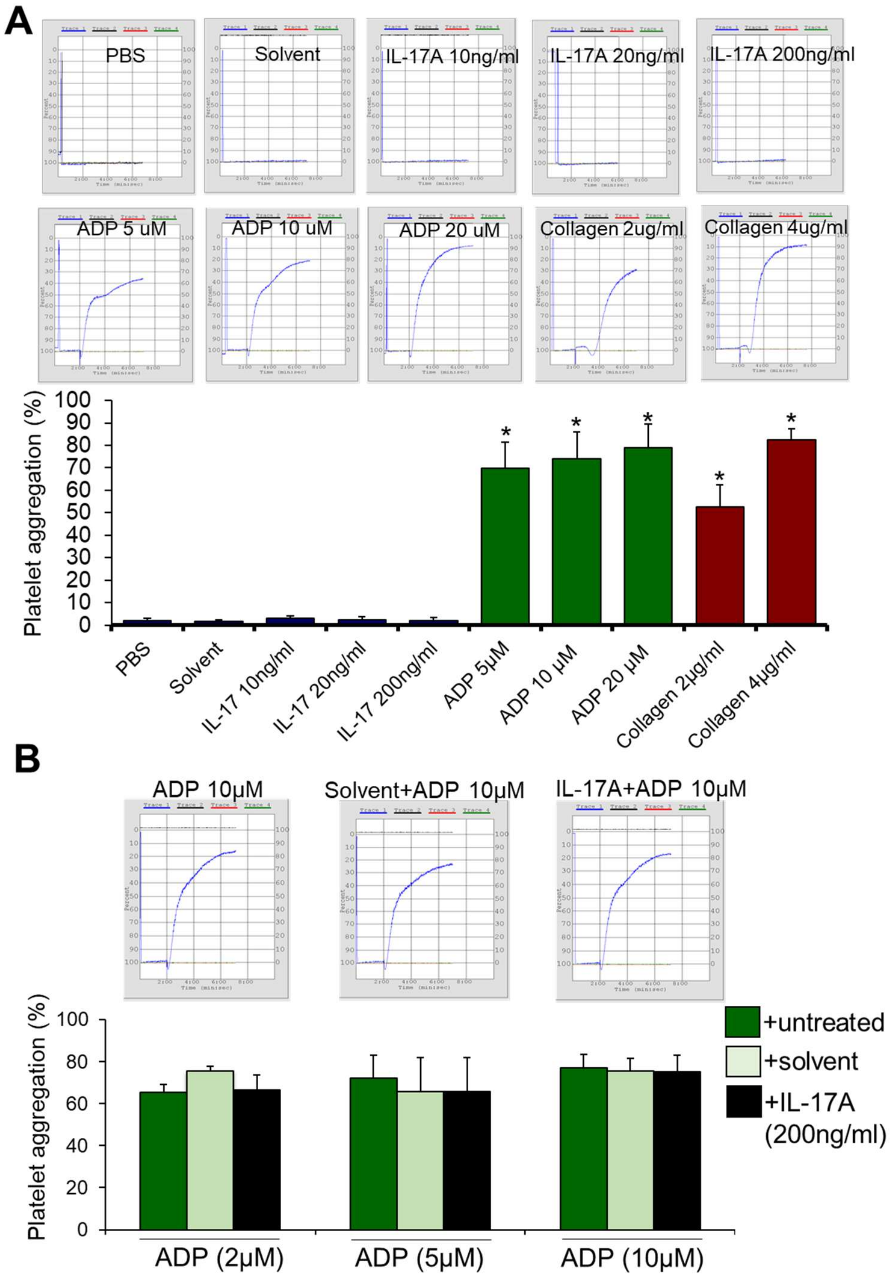


**Figure S2.** No effect of IL-17A alone in different doses or in combination with different doses of ADP in platelet aggregation. (**A**) Aggregation of platelets stimulated with various doses of IL-17A (10, 20, 200 ng/ml), ADP (5, 10, 20 μM) or collagen (2, 4 μg/ml) for 2 min at 37$^o$C before aggregation is recorded. Upper panel includes the representative images from the platelet aggregation experiments using light transmission aggregometry and quantification as percentage of aggregation (%) documented in three independent experiments (lower panel). (**B**) Aggregation of platelets stimulated with various doses of ADP (2, 5, 10 μM) and/or a high dose of IL-17A (200ng/ml). Representative images from the platelet aggregation experiments using light transmission aggregometry are depicted in the upper panel and quantification as percentage of aggregation (%) documented in three independent experiments (lower panel). Data are presented as mean±SEM. No statistical differences are present.

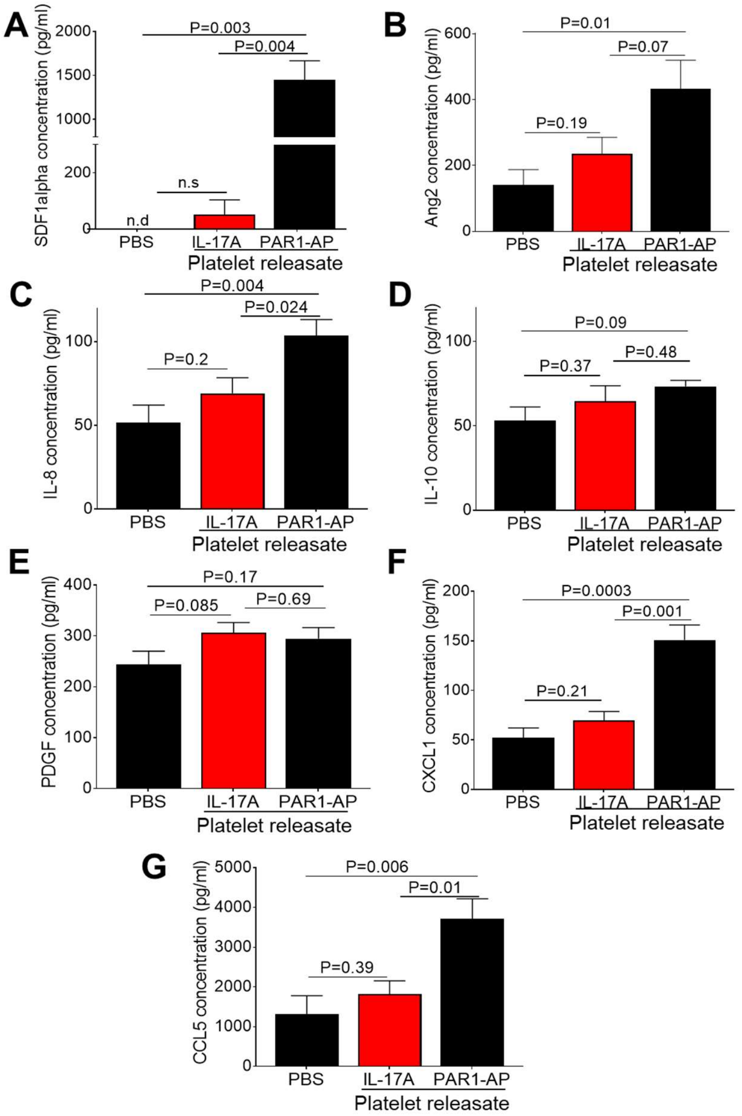


**Figure S3.** Pro- and anti- inflammatory factors that do not exhibit different levels in the platelet releasate following IL-17A stimulation of platelets. Washed platelets were incubated with IL-17A (50ng/ml) or PAR1-AP (100μM) or PBS (resting platelets) for 10 minutes at 37°C. Using ELISA, the recovered releasates were then analyzed for A) stromal derived factor alpha (SDF1$\alpha$), B) angiopoietin 2 (Ang2), C) interleukin-8 (IL-8), D) the anti-angiogenic, interleukin-10 (IL-10), E) platelet derived growth factor (PDGF), F) CXC motif chemokine ligand 1 (CXCL1), G) CC motif chemokine ligand 5 (CCL5). Values are presented as the mean concentration (pg/ml) ±SEM of six independent experiments. With regards to statistics, P-value of two-tailed t test is reported per indicated pairwise comparison as normality test was passed. No statistical significance is denoted as n.s. No detectable levels are denoted as n.d.

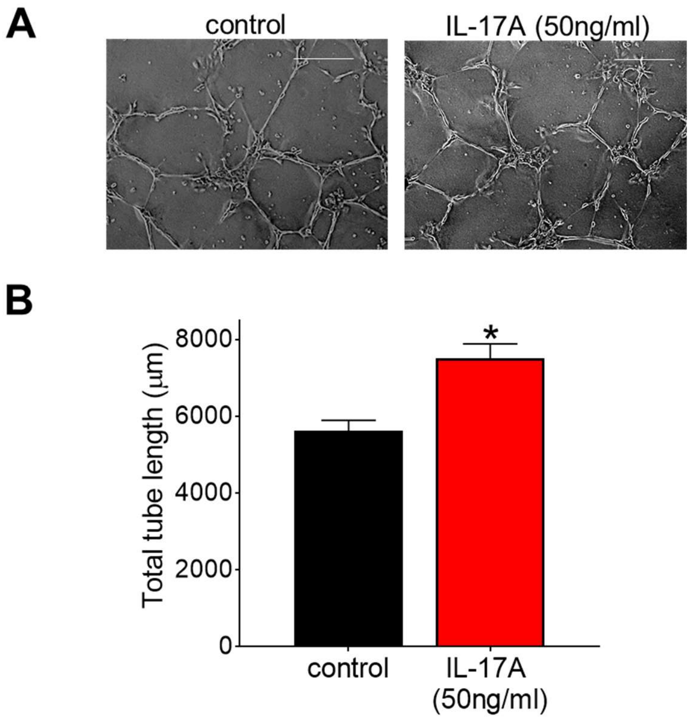


**Figure S4.** Direct effect of IL-17A on capillary-like tube formation of endothelial cells**.** (**A**) Representative images from the capillary tube formation in Matrigel-coated wells following exposure of human umbilical vein endothelial cells (HUVECs) to IL-17A (50 ng/ml) or PBS (control) for 18 hours. (**B**) Quantification of cumulative tube length was determined in 10 non-overlapping fields. Values are presented as the mean of total tube length (μm) ±SEM of three independent experiments. With regards to statistics, P-value was determined by a two-tailed t test. Scale bars indicate 500 μm.
